# Characteristics of Ambient Volatile Organic Compounds (VOCs) Measured in Shanghai, China

**DOI:** 10.3390/s100807843

**Published:** 2010-08-20

**Authors:** Chang-Jie Cai, Fu-Hai Geng, Xue-Xi Tie, Qiong Yu, Li Peng, Guang-Qiang Zhou

**Affiliations:** 1 Shanghai Meteorological Bureau, 166 Puxi Road, Shanghai 200135, China; E-Mails: changjie.cai@yahoo.com.cn (C.-J.C.); wgejgyiy@163.com (Q.Y); pengli.tulip@gmail.com (L.P.); zhougq21@163.com (G.-Q.Z.); 2 Key Laboratory for Atmospheric Physics & Environment, Nanjing University of Information Science and Technology, 219 Ningliu Road, Pukou District, Nanjing 210044, China; 3 National Center for Atmospheric Research, 1850 Table Mesa Dr., Boulder, CO 80307, USA; E-Mail: xxtie@ucar.edu (X.-X.T.)

**Keywords:** volatile organic compounds (VOCs), seasonal variation, ozone formation potential (OFP), diurnal variation, weekend effect

## Abstract

To better understand the characteristics of ambient abundance of volatile organic compounds (VOCs) in Shanghai, one of the biggest metropolis of China, VOCs were measured with a gas chromatography system equipped with a mass-selective detector (GC/MSD) from July 2006 to February 2010. An intensive measurement campaign was conducted (eight samples per day with a 3 hour interval) during May 2009. The comparison of ambient VOCs collected in different regions of Shanghai shows that the concentrations are slightly higher in the busy commercial area (28.9 ppbv at Xujiaui) than in the urban administrative area (24.3 ppbv at Pudong). However, during the intensive measurement period, the concentrations in the large steel industrial area (28.7 ppbv at Baoshan) were much higher than in the urban administrative area (18 ppbv at Pudong), especially for alkanes, alkenes, and toluene. The seasonal variations of ambient VOC concentrations measured at the Xujiahui sampling site indicate that the VOC concentrations are significantly affected by meteorological conditions (such as wind direction and precipitation). In addition, although alkanes are the most abundant VOCs at the Xujiahui measurement site, the most important VOCs contributing to ozone formation potential (OFP) are aromatics, accounting for 57% of the total OFP. The diurnal variations of VOC concentrations show that VOC concentrations are higher on weekdays than in weekends at the Xujiahui sampling site, suggesting that traffic condition and human activities have important impacts on VOC emissions in Shanghai. The evidence also shows that the major sources of isoprene are mainly resulted from gasoline evaporation at a particular time (06:00–09:00) in the busy commercial area. The results gained from this study provide useful information for better understanding the characteristics of ambient VOCs and the sources of VOCs in Shanghai.

## 1. Introduction

The Yangtze River Delta (YRD) located in the eastern China coast is the largest economic region in China, and Shanghai is the largest city in the YRD region. In the past two decades, Shanghai has undergone a rapid increase in economic development. For example, the Gross Domestic Production (GDP) is over 1.49 trillion RMB, accounting for about 21% of the total GDP in the YRD region. Industrial Gross Output (IGO) increased from 0.51 to 2.56 trillion RMB from 1996 to 2008, and the number of automobiles increased from 0.47 to 2.61 million between 1996 and 2008 [1,2]. Accompanying the rapid economic development, in recent years the air quality has deteriorated in the YRD region. For example, high particular matter (PM) concentrations and poor visibility occur [3,4]. The concentrations of O_3_ are increasing and could be another important atmospheric pollutant in the YRD region [5–7]. Thus, to better understand the characteristics of precursors of O_3_ has become an important issue for studying ozone formation and for implementing effective O_3_ control strategies in Shanghai. Some progress has been made during the past. For example, Geng *et al*. [5,6,8,9] and Tang *et al*. [10] reported that O_3_ chemical production is limited by the concentrations of VOCs (VOC-sensitive regime) in Shanghai, and different VOC species (e.g., aromatics, alkenes, alkanes, *etc.*) have different contributions to the ozone formation. In this study, intensive VOC measurements during 2006 to 2010 in different regions of Shanghai are analyzed to better quantify the characteristics of VOCs in the city.

In this study, some general knowledge related to VOC sources was applied, including the locations of industrial complexes and the meteorological conditions (prevailing wind direction) around the sampling sites. To better understand the diurnal variations of VOC concentrations, the diurnal variation of traffic flow (15 main roads) are considered in the VOC measurements. Furthermore, the OH reactivity of VOCs, which is closely related to ozone photochemical formation, is calculated. The VOC reactivity is an important factor to determine the ozone formation due to various VOC species in large cities [11–13]. In this study, a propylene-equivalent concentration method suggested by Chameides *et al*. [11] and a maximum incremental reactivity (MIR) method proposed by Carter [14] are used to calculate the OH reactivity and maximum ozone-forming potential of VOCs.

The paper is organized as the follows. In Section 2, we describe the experimental method; including the instruments, measurements, the propylene-equivalent concentration and MIR method. In Section 3, the results from measurements and calculations are discussed. The summary of the results are given in Section 4.

## 2. Experimental Method

### 2.1. GC/MSD Measurement System

**Figure 1.**
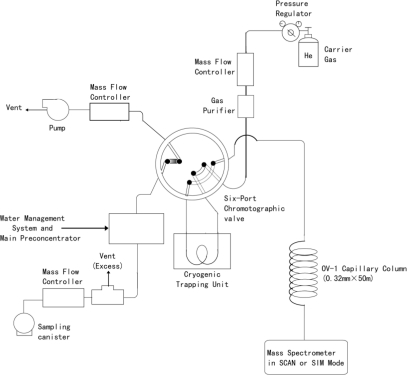
Schematic diagram of the Gas Chromatography/Mass-Selective Detection (GC/MSD) system.

In order to study the diurnal variations of VOC species, VOCs were intensively measured (eight samples per day) in different areas during 2009. VOC samples were also collected at the Xujiahui measurement site for three hours (from 6:00 to 9:00) from July 2006 to February 2010 using a 6 L Silonite canister equipped with a valve (model 29-10622, Entech Instruments Inc., USA). The internal Silonite coating improves long-term VOC storage, and the canisters have a large volume to provide detection of volatile chemicals down to low pptv range. These canisters meet or exceed the technical specifications of the US EPA method. The automatic VOC measurement system used in this study is shown in Figure 1.

Gas samples were pre-processed using a Model 7100 VOC pre-concentrator (Entech Instruments Inc., USA) and analyzed by a gas chromatography system (Agilent GC6890) equipped with a mass-selective detector (Agilent MSD5975N) with the capillary of 60 m length, 0.32 mm diameter, and a film thickness of 1.0 μm. The programmed temperature profile was used with helium as carrier gas and a flow rate of 1.5 mL·min^−1^. The column temperature was controlled at an initial temperature of −50 °C for 3 minutes, increasing it to 170 °C at the rate of 4 °C·min^−1^, and then switching to 220 °C at the rate of 14 °C·min^−1^. VOC calibration standard samples were prepared by diluting 1.0 ppm standard gas mixtures with pure nitrogen gas with an Entech 4600 Dynamic Diluter. The relative standard deviation (RSD) of the relative response factor (RRF) for most VOCs ranges from 1.5 to 11.6%. The relative error for nine measurements (accuracy) ranged from 3.7 to 19%, and the precision for seven parallel samples ranged from 1.8 to 13.6%.

## 2.2. Calibration

**Figure 2.**
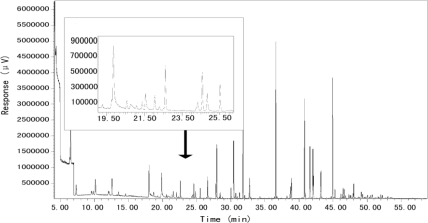
Typical chromatogram of total VOCs obtained from 06:00 to 09:00 on 2 December 2009 at the Xujiahui sampling site.

The internal standard gases were set to four concentrations (*i.e.*, 0.5 ppbv, 2.5 ppbv, 5.0 ppbv, and 10.0 ppbv) in order to get the relative response values (*μV*). The VOC species which were detected in Xujiahui are listed in Table 1. The result suggests that the linear correlation coefficients of the calibration curves (column 4 of Table 1) are quite high. The detection limits (DL) of most VOCs were calculated for a sample volume of 800 mL (column 5 of Table 1). The DL values are presented in terms of absolute mass (ng) so that the sensitivities can be compared with other different analytical systems. Figure 2 presents a typical chromatogram obtained with this measurement system. Various VOCs appear at different times, and most VOCs were clearly separated. However, acetone and pentane are excluded since the residual times of acetone and pentane are very close (19.912 for acetone and 19.982 for pentane) and the representative ion of both species appears at m/z 43, which leads to inaccurate quantification. In addition, *m*-xylene and *p*-xylene cannot be separated, and as a result, they are reported as *m/p*-xylene species.

### 2.3. Sampling Site

**Figure 3.**
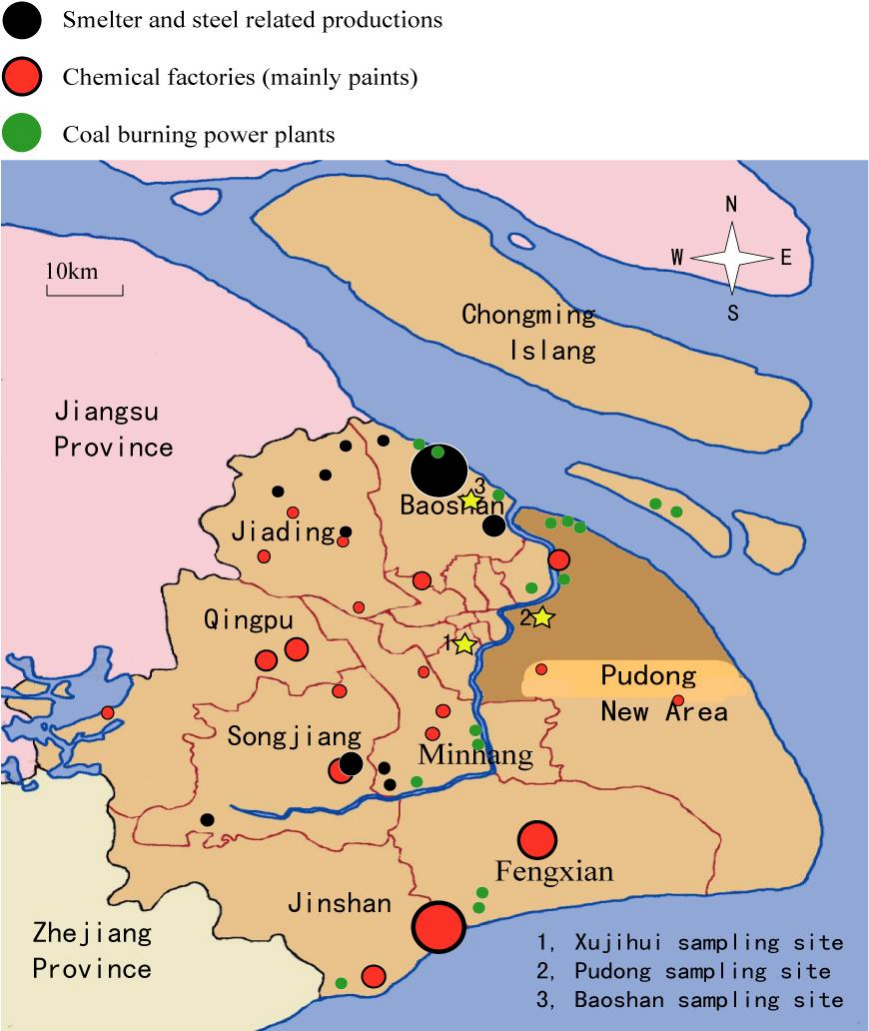
Locations of the sampling sites (stars) and the possible pollutant sources in Shanghai (different colors indicate different pollutant sources).

The three sampling sites (yellow stars in Figure 3) are located in the Xujiahui, Pudong, and Baoshan districts in the center, east and north areas of Shanghai, respectively. The Xujiahui sampling site is located in a busy commercial area. The Pudong sampling site is located in an urban administrative and office area, and the Baoshan sampling site is located in a large steel industry area. In order to gain insight into the influence of industrial factories on the three sampling sites, the distribution of industrial sites (smelters, steel, chemical factories, and coal burning power plants) in the surrounding area of Shanghai is shown in Figure 3, where it can be seen that the large smelters and steel factories are mainly located in the Baoshan and Jiading districts, which are in the north and northwest of Shanghai, the main chemical industry complex is mainly located in the west, southwest, and south regions of Shanghai, and the coal burning power plants are mainly located in the north, middle and south regions of Shanghai. Thus, wind directions become a crucial factor in controlling the VOC concentrations measured at the sampling sites, as will be discussed in Section 3.2.

### 2.4. Analysis Method

In this study, the different VOC compounds related to ozone formation were also studied according to the propylene-equivalent concentration and the MIR method. The propylene-equivalent concentration method was proposed by Chameides *et al*. [11], and the following equation is used to calculate the propylene-equivalent concentration for each individual VOC:

(1) $$\mathrm{Propy}-\mathrm{equiv}(i)=\mathrm{conc}(i)\times k_{\mathrm{OH}}(i)/k_{\mathrm{OH}}(C_{3}H_{6})$$

where *Propy-equiv(i)* is defined as a VOC compound *i* on an OH reactivity-based scale, which is normalized to the reactivity of propylene; *conc(i)* is the concentration of a VOC compound *i*; and *k_OH_(i)* is the rate constant for the reactivity of VOC compound *i* with OH radical; and *k_OH_(C_3_H_6_)* is the rate constant for the reaction of C_3_H_6_ with OH radical. The rate constants were given by Atkinson and Arey [15].

The MIR, proposed by Carter, is a good indicator for deteriming the ozone formation potential of each individual VOC species. The scenarios of this method represent conditions when ozone formation is under VOC limited conditions as suggested by several studies in the Shanghai region [5,6,8,10]. The MIR method is defined by the following equation:

(2) $$\mathrm{OFP}(i)=\mathrm{conc}(i)\times {\mathrm{MIR}}_{\mathrm{coefficient}}(i)$$

where *OFP(i)* is defined as the ozone formation potential of individual hydrocarbon *i* and *MIR_coefficient_(i)* the maximum incremental reactivity coefficient of compound *i*, which is defined by Carter [14].

## 3. Results and Discussion

### 3.1. Characteristics of Ambient VOC Concentrations in Shanghai

**Figure 4.**
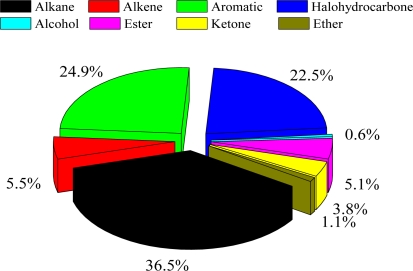
Measured VOCs in the morning (06:00–09:00) for different VOC groups (alkane, alkene, aromatic, halohydrocarbone, alcohol, ester, ketone, and ether) during July 2007–February 2010.

Table 1 summarized the measured VOC concentrations from August 2006 to February 2010 at the Xujiahui sampling site. The result indicates that the average total VOC concentration is about 41 ppbv, in which alkanes, alkenes, aromatics, and halohydrocarbons account for 36.5, 5.5, 24.9, and 22.5% of the total VOCs, respectively (see Figure 4). Other VOC species (including alcohol, ester, and ether) accounted for about 10.6% of the total VOC concentration. For individual species, toluene and propane have the highest concentrations, with average values of 4.62 and 4.56 ppbv, respectively.

**Figure 5.**
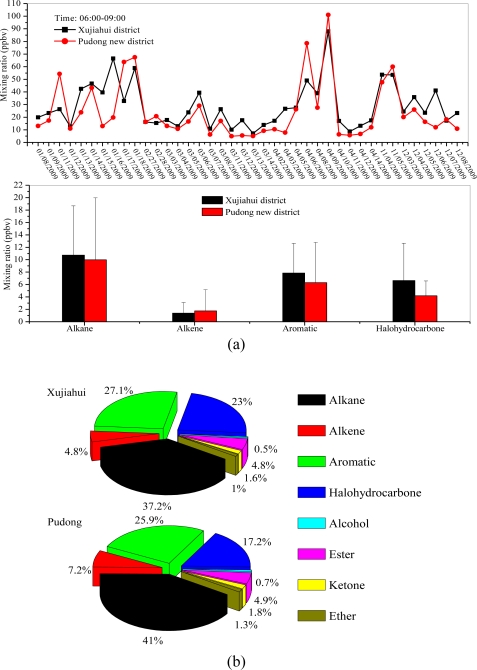
(a) Comparison of the total VOC concentrations in the morning (06:00–09:00) at two sampling sites (Xujiahui and Pudong) during 2009; (b) The measured VOC in the morning (06:00–09:00) for different VOC groups (alkanes, alkenes, aromatics, halohydrocarbons, alcohols, esters, ketones, and ethers) at two sampling sites (Xujiahui and Pudong) during 2009.

Figure 5a shows the comparison of ambient VOC concentrations measured at different sampling sites (Xujiahui and Pudong) in Shanghai during 2009. The measured VOC concentrations at the two sampling sites are listed in Table 2. The result shows that the total VOC concentrations measured at the Xujiahui site (28.9 ppbv) were slightly higher than those measured at the Pudong site (24.4 ppbv). For different VOC species, the concentrations of alkanes, aromatics, and halohydrocarbons measured at the Xujiahui site were higher than those measured at the Pudong site. However, the concentrations of alkenes measured at Xujiahui site were lower than at the Pudong site. The detailed compositions of VOC concentrations at two sampling sites are illustrated in Figure 5b. The resulta indicate that alkanes, aromatics and halohydrocarbons were the dominant VOC groups at the two sampling sites.

**Figure 6.**
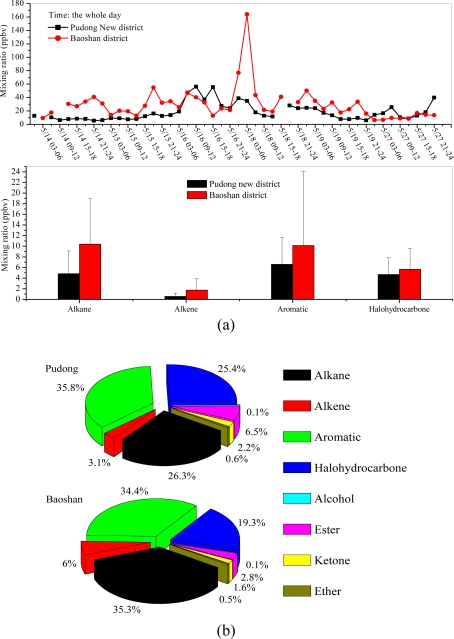
(a) Comparison of the total VOC concentrations during the whole day (8 samples per day) at 2 sampling sites (Pudong and Baoshan) during May, 2009 (b) The measured VOC during the whole day for different VOC groups (alkane, alkene, aromatic, halohydrocarbone, alcohol, ester, ketone, and ether) at 2 sampling sites (Pudong and Baoshan) during May, 2009.

The Baoshan sampling site is located in a large steel industry area in Shanghai (Figure 3). At the Baoshan site as well as the Xujiahui site, diurnal variation of VOCs was sampled (eight samples per day with a three hour interval) during May 2009. The resulting comparison of ambient VOC concentrations is shown in Figure 6a.

The measured VOC concentrations at the two sampling sites are listed in Table 3. The concentrations of alkanes, alkenes and toluene measured at the Baoshan site are considerably higher than those at the Pudong site, indicating that alkane, alkene and toluene emissions might be associated with steel production. This result is consistent with the measurements by Liu *et al.* [16], who reported that toluene concentrations are high in steel production areas. In addition, a high 1,2-dichloroethane concentration was detected which could be emitted from coal burning due to the large consumption of coal in Shanghai [9]. Otherwise, the compositions of VOCs measured in the two sampling sites are very different (see Figure 6b). The percentage of alkanes and alkenes are much higher in the Baoshan site (35.3% for alkanes and 6% for alkenes) than in the Pudong site (26.3% for alkanes and 3.1% for alkenes).

The general characteristics of the measured VOC concentrations in Shanghai were compared to similar measurements in different large cities (Table 4). The table shows the mean concentrations of VOCs measured in other important Asian cities (Seoul and Nagoya) and other metropolis in China (Beijing, Guangzhou, and Hong Kong) [17–21]. The comparison shows that the concentrations of VOCs in Shanghai are similar to those measured in Nagoya, but are much lower than those measured in Beijing, Guangzhou, Hong Kong and Seoul.

### 3.2. Seasonal Variations and Ozone Formation Potential

**Figure 7.**
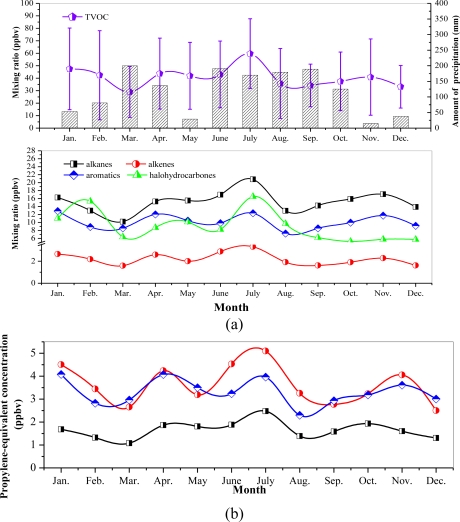
(a) Measured seasonal variations of VOC concentrations at the Xujiahui site averaged from 2006 to 2010. (b) Seasonal variations of VOC propylene-equivalent concentrations (ppbC).

**Figure 8.**
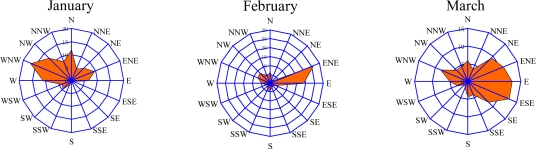

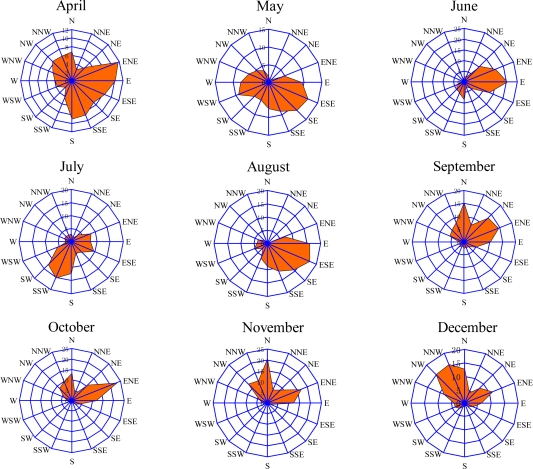
Rose diagrams of wind directions in different months at the Xujiahui site.

The seasonal variations of VOC concentrations and monthly averaged precipitation at the Xujiahui site are shown in Figure 7a. The result suggests that the VOC concentrations were lower when the precipitation was higher, except in summer. During summer, the averaged VOC concentration in July (60 ppbv) was higher than in June (43 ppbv) and August (36 ppbv). The wind directions in different months (Figure 8) show that the prevailing wind direction might have an important impact on the measured VOC concentrations. Figure 8 shows that in June and August, the prevailing wind directions were east and southeast. In this case, there were no major pollutant sources which have significant effect on the measured VOC concentration in the sampling sites. However, in July, the prevailing wind directions were south and southwest, and many major large industrial areas could have important effects on the measured VOC concentrations at the sampling sites.

Among the four VOC groups, alkanes were the dominant species throughout the year, except for February, and the seasonal variation was similar to that of total VOCs [*i.e.*, higher in July (21 ppbv) and lower in March (10 ppbv)]. The seasonal variation of aromatics and alkenes was also similar to that of total VOCs. However, the seasonal variation of halohydrocarbons was different from other VOC groups, indicating that the sources of halohydrocarbons must be different from those of other VOCs.

**Figure 9.**
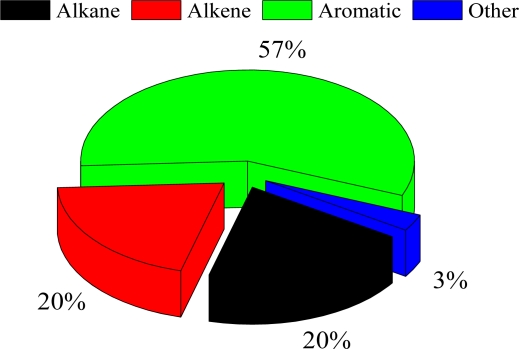
Contributions to ozone formation by different VOC groups (alkanes, alkenes, aromatics, and others).

Figure 7b also indicates that the propylene-equivalent concentrations of alkanes were lower than for aromatics and alkenes throughout the year. The propylene-equivalent concentration of alkenes reached its peak value in July (5.10 ppbv). The seasonal variation of alkenes was similar to that of aromatics. In this study, the MIR method was used to evaluate the contributions of VOC groups to ozone formation (see Figure 9). The result shows that aromatics play important roles in ozone formation, contributing to ozone formation by about 57%. This result suggests that even though the measured concentrations of alkanes were the highest, their contributions to ozone formation (20%) were less than those of aromatics, due to the lower OH reactivity.

### 3.3. Diurnal Variations and Weekend Effect

**Figure 10.**
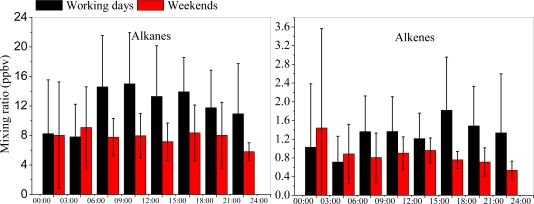

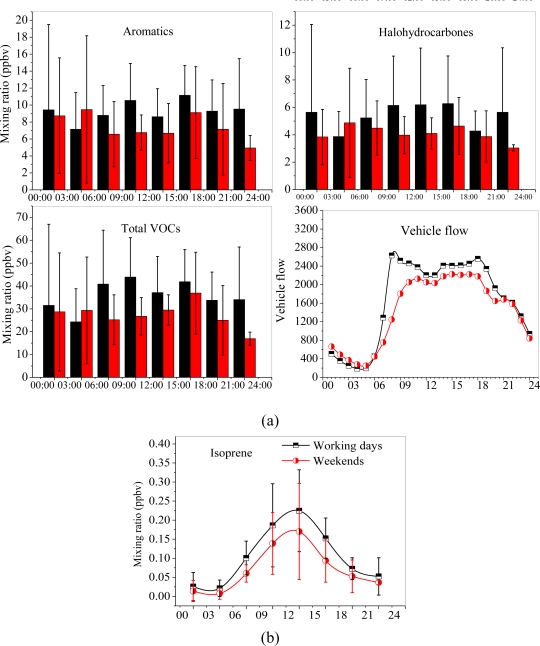
(a) Diurnal variations of VOC grouping concentrations measured at Xujiahui during weekdays (black) and weekends (red) from 25 August to 16 September, 2009. (b) Same to Figure 10a, except for isoprene.

The diurnal variations of ambient VOC concentrations from 25 August to 16 September are shown in Figure 10. The result suggests that the VOC concentrations are higher on weekdays (Monday to Friday) than in weekends (Saturday and Sunday), indicating that human activities have significant impact on the VOC emissions in Shanghai. The diurnal cycles of alkanes, alkenes and aromatics had a double-peak pattern on weekdays, especially during high traffic time (07:00–19:00) (shown in Figure 10). The two peaks occurred around 09:00 and 15:00–18:00, and were correlated to the morning and afternoon rush hours. In addition, in weekends, no clear double-peak patterns were indicated, which might due to the change in traffic pattern during the weekends. However, the diurnal variation pattern of halohydrocarbones was different with other VOCs, indicating that the different sources of halohydrocarbones.

**Figure 11.**
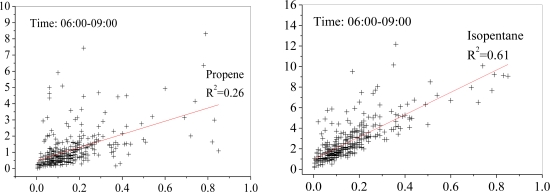
Correlations between isoprene (ppbv) and propene/isopentane (ppbv) in the morning (06:00–09:00) at the Xujiahui site.

There are two possible sources for isoprene. One is production by biogenic sources, which is normally considered as the major source of isoprene. Another possible source is from anthropogenic activity, especially in large cities [22]. Because biogenic isoprene emissions are very weak in winter, the concentration of isoprene from biogenic sources should be much lower in winter than in summer [23,24]. However, the measured isoprene concentrations in the Xujiahui site during 06:00–09:00 had a small variation in summer (0.15 ± 0.11 ppbv) and in winter (0.13 ± 0.17 ppbv). In addition, there is very little vegetation cover near the sampling site, consequently producing small amounts of the local isoprene emissions. Furthermore, the correlation coefficients among isoprene, propene and isopentane were calculated (see Figure 11). Because isopentane is a typical tracer for gasoline evaporation, the high correlation (*R^2^* = 0.61) between isoprene and isopentane suggests that isoprene was mainly related to industrial activities near the sampling site.

## 4. Conclusions

Over the past two decades, Shanghai has undergone a rapid increase in economic development. Accompanying this development, the air quality in Shanghai has deteriorated in recent years. One of the major pollution problems is that ozone concentrations are increasing and it could become an important pollutant in the region. Because VOCs are important precursors for ozone formation, understanding the characteristics and the contributions of VOCs to ozone formation is an important issue for better understanding the ozone production in Shanghai. In this study, based on the measured VOC concentrations in Shanghai from 2006 to 2010, the characterization of these VOCs, the seasonal/diurnal variations of VOCs, and their contributions to ozone formation are compared and analyzed for different sampling sites.

The results show that the seasonal variations of VOC concentrations are greatly influenced by meteorological conditions, including precipitation and wind direction. Aromatics play the most important role in ozone formation, although the concentrations of alkanes are the highest. The weekend effect shows that VOC concentrations are much higher on weekdays than on weekends, indicating that traffic conditions and human activities greatly affect VOC emissions at the sampling sites. The analysis of isoprene variation suggests that isoprene was mainly emitted from gasoline evaporation at the center of the city. The results gained from this study provide useful information for better understanding the characteristics of ambient VOCs in Shanghai.

## Figures and Tables

**Table 1.** The detailed information of measured VOC in the Xujiahui site averaged from 2006 to 2010 (495 air samples).

**Number**	**Name**	**Chemical group**	*Correlation coefficient	*DL (ng)	**Mixing ratio (ppbv)**	
					**Mean ± S.D.**	**Rang**
1	Propene	Alkene	0.999440	0.33	0.96 ± 1.03	0.00–8.31
2	1-Butene	Alkene	0.999550	0.38	0.27 ± 0.27	0.00–2.30
3	1,3-Butadiene	Alkene	0.998762	0.26	0.16 ± 0.45	0.00–8.32
4	*cis*-2-Butene	Alkene	0.999607	0.44	0.22 ± 0.25	0.00–1.75
5	*trans*-2-Butene	Alkene	0.999834	0.38	0.24 ± 0.29	0.00–2.08
6	1-Pentene	Alkene	0.999619	0.71	0.14 ± 0.11	0.00–0.70
7	Isoprene	Alkene	0.999373	0.18	0.13 ± 0.14	0.00–1.00
8	2-Pentene	Alkene	0.999566	0.23	0.12 ± 0.14	0.00–1.69
9	1-Hexene	Alkene	0.999467	0.66	0.03 ± 0.07	0.00–0.80
10	Propane	Alkane	0.999509	0.20	4.56 ± 2.71	0.00–21.88
11	Isobutane	Alkane	0.999797	0.27	1.41 ± 1.09	0.07–6.74
12	Butane	Alkane	0.999780	0.17	2.08 ± 1.40	0.23–9.28
13	Isopentane	Alkane	0.999637	0.56	2.36 ± 1.77	0.13–12.17
14	2,2-Dimethylbutane	Alkane	0.999736	0.45	0.03 ± 0.06	0.00–0.95
15	Cyclopentane	Alkane	0.998352	0.34	0.10 ± 0.17	0.00–3.27
16	2,3-Dimethylbutane	Alkane	0.999659	0.39	0.14 ± 0.18	0.00–2.48
17	2-Methylpentane	Alkane	0.999641	0.48	0.80 ± 1.18	0.04–15.57
18	3-Methylpentane	Alkane	0.999741	0.48	0.60 ± 1.12	0.02–15.57
19	*n*-Hexane	Alkane	0.999956	0.76	0.97 ± 1.66	0.03–24.25
20	2,4-Dimethylpentane	Alkane	0.999749	0.42	0.23 ± 0.27	0.01–3.50
21	Methylcyclopentane	Alkane	0.999516	0.38	0.28 ± 0.34	0.00–4.72
22	Cyclohexane	Alkane	0.999238	0.41	0.11 ± 0.11	0.00–0.93
23	2-Methylhexane	Alkane	0.997083	0.42	0.20 ± 0.20	0.00–1.92
24	2,3-Dimethylpentane	Alkane	0.999302	0.49	0.09 ± 0.09	0.00–0.69
25	3-Methylhexane	Alkane	0.998441	0.46	0.21 ± 0.22	0.00–1.86
26	2,2,4-Trimethylpentane	Alkane	0.997326	0.63	0.03 ± 0.06	0.00–0.96
27	*n*-Heptane	Alkane	0.994699	0.59	0.22 ± 0.20	0.00–1.42
28	Methylcyclohexane	Alkane	0.999567	0.29	0.09 ± 0.08	0.00–0.79
29	2,3,4-Trimethylpentane	Alkane	0.997527	0.30	0.01 ± 0.03	0.00–0.71
30	2-Methylheptane	Alkane	0.997880	0.34	0.05 ± 0.04	0.00–0.30
31	3-Methylheptane	Alkane	0.995449	0.37	0.08 ± 0.08	0.00–0.60
32	*n*-Octane	Alkane	0.996870	0.34	0.10 ± 0.23	0.00–4.75
33	Nonane	Alkane	0.985404	0.54	0.10 ± 0.10	0.00–1.08
34	*n*-Decane	Alkane	0.994657	0.83	0.10 ± 0.10	0.00–2.49
35	*n*-Dodecane	Alkane	0.995704	2.11	0.09 ± 0.14	0.00–1.11
36	*n*-Undecane	Alkane	0.999883	2.09	0.09 ± 0.18	0.00–3.53
37	Styrene	Aromatic	0.999182	0.65	0.15 ± 0.19	0.00–2.86
38	Benzene	Aromatic	0.999608	0.46	1.76 ± 1.38	0.29–12.56
39	Toluene	Aromatic	0.998413	0.57	4.62 ± 4.52	0.35–42.35
40	Ethylbenzene	Aromatic	0.998121	0.69	1.29 ± 1.20	0.06–9.14
41	*m*-Xylene	Aromatic	0.995785	0.62	1.36 ± 1.21	0.11–8.79
42	*p*-Xylene	Aromatic		0.66		
43	*o*-Xylene	Aromatic	0.993700	0.66	0.55 ± 0.64	0.05–8.36
44	Isopropylbenzene	Aromatic	0.999219	0.35	0.08 ± 0.25	0.00–4.45
45	*n*-Propylbenzene	Aromatic	0.999922	0.31	0.07 ± 0.07	0.00–0.83
46	1,3,5-Trimethylbenzene	Aromatic	0.995870	0.86	0.06 ± 0.06	0.00–0.91
47	*m*-Ethyltoluene	Aromatic	0.999164	1.35	0.19 ± 0.19	0.02–1.19
48	*p*-Ethyltoluene	Aromatic	0.995702	0.77	0.11 ± 0.08	0.01–0.74
49	*o*-Ethyltoluene	Aromatic	0.999347	0.89	0.06 ± 0.05	0.00–0.84
50	*m*-Diethylbenzene	Aromatic	0.999796	0.96	0.02 ± 0.03	0.00–0.43
51	Vinyl chloride	Halohydrocabone	0.996365	0.39	1.07 ± 3.51	0.00–33.83
52	1,1-Dichloroethene	Halohydrocabone	0.999456	0.54	0.01 ± 0.06	0.00–1.12
53	Allyl chloride	Halohydrocabone	0.999718	0.25	0.03 ± 0.10	0.00–2.11
54	*trans*-1,2-Dichloroethene	Halohydrocabone	0.999731	0.57	0.01 ± 0.03	0.00–0.58
55	*cis*-1,2-Dichloroethene	Halohydrocabone	0.999986	0.54	0.00 ± 0.03	0.00–0.52
56	*cis*-1,3-Dichloropropene	Halohydrocabone	0.999962	0.51	0.00 ± 0.03	0.00–0.46
57	*trans*-1,3-Dichloropropene	Halohydrocabone	0.999383	0.62	0.08 ± 0.09	0.00–0.76
58	Tetrachloroethylene	Halohydrocabone	0.999676	0.87	0.19 ± 1.44	0.00–30.95
59	Hexachloro-1,3-butadiene	Halohydrocabone	0.999645	2.04	0.08 ± 0.16	0.00–0.88
60	Trichloroethylene	Halohydrocabone	0.999693	0.69	0.15 ± 0.31	0.00–3.65
61	Freon-12	Halohydrocabone	0.999827	0.63	0.58 ± 0.13	0.00–1.95
62	Chloromethane	Halohydrocabone	0.995610	0.38	1.76 ± 2.64	0.14–23.31
63	Freon-114	Halohydrocabone	0.997958	1.06	0.00 ± 0.00	0.00–0.02
64	Bromomethane	Halohydrocabone	0.998258	0.47	1.26 ± 4.41	0.00–17.95
65	Chloroethane	Halohydrocabone	0.998785	0.38	0.05 ± 0.10	0.00–1.95
66	Freon-11	Halohydrocabone	0.999825	0.81	0.31 ± 0.10	0.00–2.00
67	Freon-113	Halohydrocabone	0.997887	1.59	0.08 ± 0.03	0.00–0.44
68	Methylene chloride	Halohydrocabone	0.999810	0.67	0.95 ± 0.96	0.00–13.93
69	1,1-Dichloroethane	Halohydrocabone	0.999859	0.52	0.12 ± 0.58	0.00–8.92
70	Chloroform	Halohydrocabone	0.999752	0.62	0.15 ± 0.18	0.00–1.69
71	1,2-Dichloroethane	Halohydrocabone	0.999767	0.55	1.53 ± 2.25	0.08–23.02
72	Carbon tetrachloride	Halohydrocabone	0.999621	0.75	0.22 ± 0.94	0.00–20.45
73	1,2-Dichloropropane	Halohydrocabone	0.999582	0.55	0.18 ± 0.71	0.00–11.07
74	Bromodichloromethane	Halohydrocabone	0.999257	0.86	0.01 ± 0.01	0.00–0.03
75	1,1,2-Trichloroethane	Halohydrocabone	0.999925	0.74	0.04 ± 0.05	0.00–0.55
76	Dibromochloromethane	Halohydrocabone	0.999857	1.02	0.09 ± 0.91	0.00–19.62
77	1,2-Dibromoethane	Halohydrocabone	0.999775	0.98	0.00 ± 0.01	0.00–0.05
78	Bromoform	Halohydrocabone	0.999508	1.32	0.01 ± 0.02	0.00–0.09
79	1,1,1-Trichloroethane	Halohydrocabone	0.999943	0.57	0.02 ± 0.02	0.00–0.15
80	Tetrachloroethane	Halohydrocabone	0.999898	1.10	0.01 ± 0.03	0.00–0.20
81	Chlorobenzene	Halohydrocabone	0.999950	0.66	0.07 ± 0.08	0.00–0.54
82	1,3-Dichlorobenzene	Halohydrocabone	0.998193	0.82	0.14 ± 0.18	0.00–1.19
83	Benzyl chloride	Halohydrocabone	0.999574	0.66	0.03 ± 0.03	0.00–0.14
84	1,2-Dichlorobenzene	Halohydrocabone	0.999291	0.96	0.04 ± 0.07	0.00–0.50
85	1,2,4-Trichlorobenzene	Halohydrocabone	0.997771	1.90	0.06 ± 0.13	0.00–0.67
86	Isopropyl alcohol	Alcohol	0.999995	0.63	0.27 ± 1.08	0.00–14.32
87	Vinyl acetate	Ester	0.998669	0.48	0.12 ± 0.28	0.00–2.83
88	Ethyl acetate	Ester	0.998476	0.83	2.01 ± 2.59	0.00–15.48
89	2-Butanone	Ketone	0.999973	2.05	1.45 ± 1.80	0.00–12.42
90	Methyl isobutyl ketone	Ketone	0.998836	0.79	0.10 ± 0.12	0.00–0.83
91	Methyl butyl ketone	Ketone	0.995532	0.72	0.02 ± 0.02	0.00–0.14
92	Methyl tert-butyl ether	Ether	0.995313	0.46	0.33 ± 0.33	0.00–2.39
93	Tetrahydrofuran	Ether	0.999881	0.40	0.12 ± 0.14	0.00–1.25
	TVOCs				41.43 ± 30.13	7.12–210.87

* The correlation coefficients is for the calibrations at four concentrations (0.5, 2.5, 5.0, 10.0 ppbv); DL = detection limit.

**Table 2.** Measured VOC concentrations (ppbv) at the Xujiahui and Pudong sampling sites (sampling period: 06:00–09:00).

**Compounds**	**Xujiahui site (40 samples)**			**Pudong site (40 samples)**		
	**Mean ± S.D.**	**Median**	**Range**	**Mean ± S.D.**	**Median**	**Range**
Propene	0.66 ± 0.67	0.40	0.04–3.14	0.65 ± 1.17	0.36	0.00–7.23
1-Butene	0.14 ± 0.16	0.07	0.00–0.60	0.19 ± 0.33	0.08	0.00–1.42
*cis*-2-Butene	0.12 ± 0.16	0.06	0.00–0.95	0.20 ± 0.36	0.06	0.00–1.63
*trans*-2-Butene	0.15 ± 0.24	0.07	0.00–1.42	0.21 ± 0.40	0.05	0.00–1.72
1-Pentene	0.07 ± 0.08	0.05	0.00–0.41	0.07 ± 0.11	0.03	0.00–0.48
2-Pentene	0.06 ± 0.09	0.03	0.00–0.50	0.09 ± 0.16	0.02	0.00–0.76
Isoprene	0.05 ± 0.13	0.02	0.00–0.82	0.03 ± 0.05	0.00	0.00–0.18
Propane	3.98 ± 2.41	3.75	1.05–14.67	3.07 ± 2.76	2.67	0.07–13.49
Isobutane	1.01 ± 0.86	0.84	0.07–4.46	0.95 ± 1.15	0.60	0.00–4.36
Butane	1.62 ± 1.26	1.35	0.23–7.30	1.43 ± 1.36	1.04	0.08–5.00
Isopentane	1.53 ± 1.13	1.22	0.13–5.70	1.90 ± 2.31	1.10	0.17–9.23
2-Methyl pentane	0.43 ± 0.45	0.29	0.07–2.40	0.46 ± 0.62	0.21	0.02–3.25
3-Methyl pentane	0.33 ± 0.42	0.19	0.04–2.39	0.35 ± 0.62	0.13	0.02–3.60
*n*-Hexane	0.58 ± 0.85	0.30	0.07–5.09	0.64 ± 1.42	0.22	0.00–7.98
Styrene	0.12 ± 0.12	0.08	0.03–0.73	0.12 ± 0.19	0.06	0.00–1.15
Benzene	1.50 ± 0.99	1.22	0.40–5.03	1.13 ± 0.89	0.87	0.20–4.19
Toluene	3.21 ± 2.29	2.76	0.63–10.15	2.64 ± 3.03	1.48	0.26–11.22
Ethylbenzene	0.89 ± 0.89	0.71	0.15–4.97	0.64–0.79	0.29	0.08–3.04
*m/p*-Xylene	1.32 ± 1.05	0.96	0.13–4.43	1.08 ± 1.30	0.53	0.12–5.30
*o*-Xylene	0.36 ± 0.24	0.27	0.08–0.95	0.29 ± 0.33	0.16	0.06 ± 1.37
Chloromethane	1.62 ± 2.89	0.92	0.14–18.39	1.02 ± 0.80	0.84	0.19–4.13
Freon-12	0.56 ± 0.14	0.59	0.00–0.74	0.52 ± 0.16	0.58	0.02–0.91
Freon-11	0.28 ± 0.05	0.28	0.00–0.36	0.27 ± 0.04	0.28	0.04–0.33
Chloroform	0.12 ± 0.13	0.09	0.00–0.77	0.09 ± 0.07	0.07	0.03–0.39
1,2-Dichloroethane	1.51 ± 1.80	0.83	0.13–9.39	0.67 ± 0.79	0.36	0.10–0.45
Carbon tetrachloride	0.20 ± 0.17	0.13	0.00–0.85	0.11 ± 0.02	0.11	0.02–0.18
Ethyl acetate	1.32 ± 1.49	0.92	0.04–7.38	1.13 ± 1.80	0.38	0.02–7.49
2-Butanone	0.41 ± 0.54	0.13	0.00–2.34	0.40 ± 0.60	0.09	0.00–2.74
Methyl tert-butyl ether	0.22 ± 0.39	0.11	0.02–2.39	0.24 ± 0.42	0.06	0.00–1.96
Total VOCs	28.90 ± 17.83	23.66	7.14–87.96	24.35 ± 22.68	16.49	4.98–100.97

**Table 3.** Measured VOC concentrations (ppbv) at Baoshan and Pudong sampling sites (sampling period: 8 samples per day with a 3 hour interval).

**Compounds**	**Baoshan site (45 samples)**			**Pudong site (46 samples)**		
	**Mean ± S.D.**	**Median**	**Range**	**Mean ± S.D.**	**Median**	**Range**
Propene	0.99 ± 1.31	0.50	0.03–5.86	0.31 ± 0.38	0.13	0.03–1.97
1-Butene	0.19 ± 0.23	0.11	0.00–1.12	0.07 ± 0.09	0.03	0.00–0.35
*cis*-2-Butene	0.13 ± 0.23	0.05	0.00–0.98	0.03 ± 0.05	0.00	0.00–0.24
*trans*-2-Butene	0.14 ± 0.24	0.03	0.00–0.95	0.02 ± 0.04	0.00	0.00–0.23
1-Pentene	0.06 ± 0.06	0.04	0.00–0.25	0.03 ± 0.03	0.02	0.00–0.10
2-Pentene	0.02 ± 0.04	0.01	0.00–0.22	0.01 ± 0.01	0.00	0.00–0.04
Isoprene	0.06 ± 0.06	0.05	0.00–0.22	0.07 ± 0.09	0.04	0.00–0.42
Propane	4.21 ± 4.46	3.12	0.65–29.95	1.63 ± 1.43	1.04	0.12–5.62
Isobutane	1.00 ± 0.93	0.76	0.05–3.97	0.46 ± 0.56	0.24	0.00–2.14
Butane	1.74 ± 1.63	1.35	0.14–8.16	0.66 ± 0.68	0.37	0.06–2.72
Isopentane	1.30 ± 1.05	1.03	0.10–4.52	0.74 ± 0.65	0.47	0.10–2.81
2-Methyl pentane	0.34 ± 0.31	0.27	0.01–1.50	0.21 ± 0.22	0.13	0.04–1.05
3-Methyl pentane	0.25 ± 0.29	0.16	0.01–1.52	0.15 ± 0.21	0.09	0.02–1.25
*n*-Hexane	0.54 ± 0.67	0.34	0.02–3.28	0.31 ± 0.49	0.15	0.03–2.90
Styrene	0.10 ± 0.14	0.05	0.01–0.72	0.09 ± 0.13	0.05	0.02–0.88
Benzene	1.16 ± 1.03	0.82	0.15–4.65	0.77 ± 0.73	0.52	0.17–3.17
Toluene	5.83 ± 12.15	1.98	0.25–76.25	2.71 ± 2.43	1.94	0.43–10.15
Ethylbenzene	0.96 ± 0.67	0.81	0.14–2.68	0.98 ± 1.06	0.72	0.16–6.70
*m/p*-Xylene	1.29 ± 0.89	1.10	0.18–3.57	1.37 ± 1.58	0.94	0.19–9.38
*o*-Xylene	0.34 ± 0.25	0.29	0.06–1.40	0.35 ± 0.37	0.24	0.07–2.05
Chloromethane	1.29 ± 0.77	1.07	0.66–4.40	1.21 ± 1.26	0.85	0.26–7.68
Freon-12	0.74 ± 0.23	0.68	0.23–1.51	0.61 ± 0.15	0.62	0.15–0.90
Freon-11	0.30 ± 0.05	0.30	0.05–0.46	0.31 ± 0.06	0.31	0.06–0.51
Chloroform	0.14 ± 0.13	0.07	0.03–0.58	0.11 ± 0.11	0.05	0.03–0.55
1,2-Dichloroethane	1.15 ± 2.18	0.85	0.14–15.03	0.71 ± 0.70	0.49	0.11–3.41
Carbon tetrachloride	0.12 ± 0.02	0.12	0.02–0.20	0.12 ± 0.02	0.12	0.02–0.19
Ethyl acetate	0.78 ± 0.58	0.75	0.02–2.51	1.17 ± 1.16	0.77	0.13–4.38
2-Butanone	0.42 ± 0.35	0.36	0.01–1.51	0.35 ± 0.45	0.22	0.01–1.94
Methyl *tert*-butyl ether	0.11 ± 0.10	0.08	0.00–0.43	0.08 ± 0.09	0.05	0.00–0.32
Total VOCs	28.78 ± 25.14	23.42	6.44–164.04	18.40 ± 13.03	13.28	5.58–56.22

**Table 4.** Measured VOC concentrations (ppbv) in different urban areas.

**VOC species**	**Shanghai (XJH) China**	**Beijing China**	**Guangzhou China**	**Hong Kong (CW) China**	**Seoul Korea**	**Nagoya Japan**
	**This work**	**[17]**	**[18]**	**[19]**	**[20]**	**[21]**
Propene	0.84	1.97	1.79	1.31	2.12	0.705
1-Butene	0.26	2.21	0.60	-	0.22	0.214
*cis*-2-Butene	0.22	0.75	0.43	-	0.22	0.141
*tran*s-2-Butene	0.24	0.86	0.54	-	0.36	0.143
1-Pentene	0.13	0.34	0.33	0.52	0.11	0.147
Isoprene	0.12	1.12	0.26	0.86	0.34	0.656
Propane	4.81	6.24	5.39	3.57	9.57	3.339
Isobutane	1.43	5.36	3.11	2.75	3.16	1.404
Butane	2.03	6.36	4.60	5.55	5.37	2.661
Isopentane	2.29	11.84	3.00	3.05	2.24	1.331
2-Methyl pentane	0.67	-	1.28	-	0.14	0.371
3-Methyl pentane	0.48	-	1.02	-	0.13	0.290
*n*-Hexane	0.84	2.22	1.13	-	3.30	0.555
2,4-Dimethyl pentane	0.21	-	-	-	6.98	0.058
Methyl cyclopentane	0.27	-	0.54	-	4.38	0.128
2-Methyl hexane	0.18	-	-	-	0.43	0.121
3-Methyl hexane	0.21	-	-	-	0.44	0.143
*n*-Heptane	0.23	-	1.22	-	0.60	0.156
*n*-Nonane	0.09	-	0.22	-	1.52	0.129
*n*-Decane	0.09	1.50	-	-	2.31	0.187
Styrene	0.14	-	-	0.88	0.51	0.133
Benzene	1.81	5.43	2.80	2.11	0.84	0.519
Toluene	4.70	11.14	14.09	13.45	39.80	2.544
Ethyl benzene	1.23	4.08	2.21	1.34	4.35	0.524
*m/p*-Xylene	1.40	8.54	5.16	1.56	5.25	0.675
*o*-Xylene	0.49	3.91	2.63	0.53	2.08	0.253
MTBE	0.29	3.07	-	-	-	-
